# Regulation of the Nrf2 Pathway by Glycogen Synthase Kinase-3β in MPP^+^-Induced Cell Damage

**DOI:** 10.3390/molecules24071377

**Published:** 2019-04-08

**Authors:** Güliz Armagan, Elvin Sevgili, Fulya Tuzcu Gürkan, Fadime Aydın Köse, Tuğçe Bilgiç, Taner Dagcı, Luciano Saso

**Affiliations:** 1 Department of Biochemistry, Faculty of Pharmacy, Ege University, 35100 Bornova, Izmir, Turkey; elvinsevgili@windowslive.com (E.S.); fadime.aydin@ege.edu.tr (F.A.K.); 2 Department of Physiology, School of Medicine, Ege University, 35100 Bornova, Izmir, Turkey; fulya_tuzcu@yahoo.com (F.T.G.); tugce_bilgic@icloud.com (T.B.); tanerdagci@gmail.com (T.D.); 3 Department of Physiology and Pharmacology “Vittorio Erspamer”, Sapienza University of Rome, 00185 Rome, Italy; luciano.saso@uniroma1.it

**Keywords:** MPP^+^-induced cellular damage, glycogen synthase kinase-3β, Nrf2 pathway

## Abstract

Recently, nuclear translocation and stability of nuclear factor erythroid 2 (NF-E2)-related factor 2 (Nrf2) have gained increasing attention in the prevention of oxidative stress. The present study was aimed to evaluate the regulatory role of glycogen synthase kinase-3β (GSK-3β) inhibition by tideglusib through the Nrf2 pathway in a cellular damage model. Gene silencing (siRNA-mediated) was performed to examine the responses of Nrf2-target genes (i.e., heme oxygenase-1, NAD(P)H:quinone oxidoreductase1) to siRNA depletion of Nrf2 in MPP^+^-induced dopaminergic cell death. Nrf2 and its downstream regulated genes/proteins were analyzed using Real-time PCR and Western Blotting techniques, respectively. Moreover, free radical production, the changes in mitochondrial membrane potential, total glutathione, and glutathione-S-transferase were examined. The possible contribution of peroxisome proliferator-activated receptor gamma (PPARγ) to tideglusib-mediated neuroprotection was evaluated. The number of viable cells and mitochondrial membrane potential were increased following GSK-3β enzyme inhibition against MPP^+^. HO-1, NQO1 mRNA/protein expressions and Nrf2 nuclear translocation significantly triggered by tideglusib. Moreover, the neuroprotection by tideglusib was not observed in the presence of siRNA Nrf2. Our study supports the idea that GSK-3β enzyme inhibition may modulate the Nrf2/ARE pathway in cellular damage and the inhibitory role of tideglusib on GSK-3β along with PPARγ activation may be responsible for neuroprotection.

## 1. Introduction

Neuronal death plays a critical role in various neurodegenerative diseases characterized by the loss or dysfunction of neurons. Various risk factors such as pesticide, herbicide, industrial chemical exposure, and genetic susceptibility are among the causes of neurodegenerative diseases. The drugs and surgical interventions are currently limited and can only help to reduce the symptoms associated with disease. Thus, the development of novel therapeutic strategies to arrest and slow the onset of the disease is very important.

The common mechanisms at the cellular level, including oxidative stress, mitochondrial dysfunction, endoplasmic reticulum stress, proteasome impairment, and protein aggregation, seem to play significant roles in neurodegenerative diseases. These features affect cellular functions which lead to cell death. The importance of oxidative stress in the loss of dopaminergic neurons is well-known [1,2]. The production of reactive oxygen species (ROS) and electrophilic quinone molecules is increased inside the cell because of domaine oxidation. These species and molecules significantly lead to oxidative stress in dopaminergic neurons. In recent years, there has been an increasing focus on the link between nuclear factor erythroid 2 (NF-E2)-related factor 2/antioxidant response element (Nrf2/ARE) pathway and dopaminergic cell death [3,4,5].

The Nrf2/ARE pathway regulates cellular defense mechanisms against oxidative damage. Nrf2 is a transcription factor present in the cytoplasm. Under physiological conditions, Keap1 binds to Nrf2 and sequesters Nrf2 in the cytosol. Under oxidative stress or in the presence of Nrf2 activating compounds, Nrf2 translocates into the nucleus and regulates the expression of various ARE-responsive genes including heme oxygenase-1 (HO-1), NAD(P)H:quinone oxidoreductase 1 (NQO1), glutathione-S-transferase (GST). Thus, activation of Nrf2 is considered to be one of the protective mechanisms against the detrimential effects of oxidative stress and the drugs that inhibit the degradation of Nrf2 or induce translocation of Nrf2 into the nucleus are among the potential therapeutic alternatives in combating oxidative stress.

Glycogen synthase kinase-3beta (GSK-3β) (EC 2.7.11.26) is among the regulators of Nrf2 activation [6,7]. The activation of Fyn with phosphorylation by GSK-3β results in its nuclear accumulation. Fyn-mediated Nrf2 phosphorylation at Tyr568 or phosphorylation of Nrf2 at serine residues directly by GSK-3β leads to degradation of Nrf2 that diminishes its protection [6,8]. GSK-3β regulates distinct cellular functions including proliferation, differentiation, metabolism, and apoptosis. In pathological conditions such as cancer, inflammation, and neurodegenerative disorders, the activity of this enzyme is increased [9,10,11,12,13] and phosphorylation at different residues and inhibition by endogenous peptides regulate its basal activity [14].

Current studies on GSK-3β enzyme inhibitors focus on the treatment of neurodegenerative diseases. Specific inhibitors have been shown to inhibit 1-methyl-4-phenyl-1,2,3,6-tetrahydropyridine (MPTP)-induced apoptosis [12]. Tideglusib is both a GSK-3β non-ATP competitive inhibitor [15,16] and an agonist of peroxisome proliferator-activated receptor gamma (PPARγ) [13,17]. The therapeutic potential of this thiadiazolidinone (TDZD) compound has been evaluated in progressive supranuclear palsy which is often confused with PD, and it is reported to reduce brain atrophy [18]. Moreover, significant reduction in the characteristic symptoms of Alzheimer’s pathology such as β-amyloid plaque accumulation and tau hyperphosphorylation with tideglusib treatment was shown in transgenic mouse model [19]. In our previous study, evidence supporting its neuroprotection was obtained in NMDA/D-serine-induced cell death [13].

In the current study, it is aimed to evaluate the effect of tideglusib in 1-methyl-4-phenylpyridinium ion (MPP^+^)-induced dopaminergic neuronal death because of its potential role in regulating Nrf2. Various cellular mechanisms including free radical generation, mitochondrial membrane potential (MMP) and antioxidant enzyme activities were examined. Moreover, the changes in Nrf2-dependent antioxidant and cytoprotective genes were analyzed at mRNA and protein levels by Real-time PCR and Western blotting, respectively. In addition, siRNA-mediated gene silencing was performed to evaluate the cellular viability and the response of Nrf2-target genes to siRNA depletion of Nrf2 in our experimental model.

## 2. Results

### 2.1. Tideglusib and Pioglitazone Significantly Increased Cell Viability in MPP^+^-Treated Cells.

MPP^+^ is a widely used toxin for inducing PD-like models in in vitro studies. IC_50_ value of MPP^+^ was investigated and found as 2 mM in our previous study and adopted to present study [20]. Firstly, cells were treated with various concentrations of tideglusib or pioglitazone at three different time points (12, 24, 48 h) in order to demonstrate that tested concentrations are not toxic (data not shown). Pioglitazone is a well-known PPARγ agonist. Thus, pioglitazone was chosen to compare the effectiveness of tideglusib while investigating PPARγ-mediated neuroprotection.

**Figure 1:**
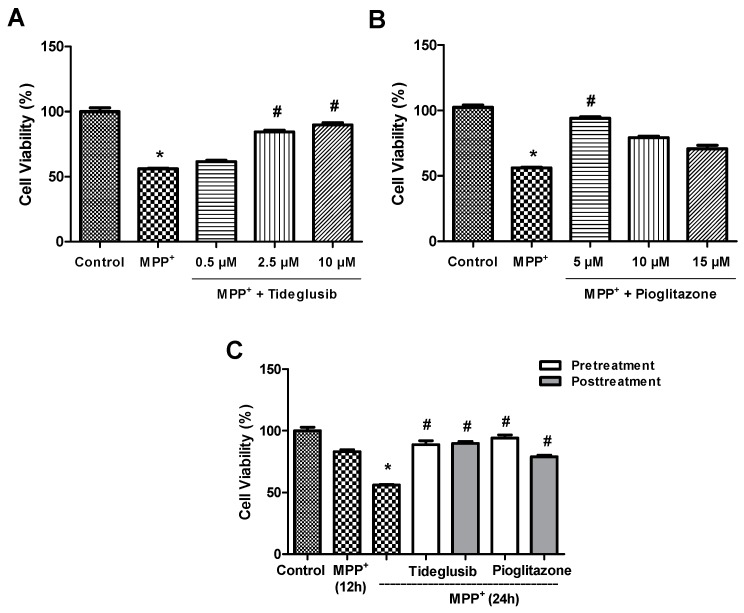
Cell viability analysis following tideglusib and pioglitazone treatments against MPP^+^-induced toxicity. Cells were pretreated with various concentrations of (**A**) tideglusib (0.5, 2.5, 10 µM) or (**B**) pioglitazone (5, 10, 15 µM) for 1 h and exposed to MPP^+^ for 24 h. (**C**) For pretreatment, cells were pretreated with tideglusib (2.5 µM) or pioglitazone (5 µM) for 1 h and exposed to MPP^+^ (2 mM) for 24 h. For post-treatment, cells were treated with MPP^+^ (2 mM) for 12 h, followed by the post-treatment of drugs for another 12 h. Cell viability was measured by MTT assay. * *p* ≤ 0.01 vs. untreated cells, ^#^ *p* ≤ 0.05 vs. MPP^+^ (24 h)-treated cells.

Tideglusib or pioglitazone pretreatments were tested with indicated concentrations (0.5, 2.5, and 10 μM for tideglusib; 5, 10, and 15 µM for pioglitazone) at 24 h against MPP^+^ and the concentrations for neuroprotection were found as 2.5 μM and 5 µM, respectively (Figure 1A,B). In order to determine the exposure time of drugs, two different protocols, pre- and post-treatment, were evaluated. For pretreatment, cells were pretreated with tideglusib (2.5 µM) or pioglitazone (5 µM) for 1 h and then exposed to MPP^+^ (2 mM) for 24 h. For post-treatment, cells were treated with MPP^+^ (2 mM) for 12 h, followed by the post-treatment of drugs for another 12 h. Both pre- and post-treatments with tideglusib significantly reduced MPP^+^-induced toxicity (pre- 88.75 ± 7.11%; post- 89.82 ± 3.69%, *p* < 0.05) (Figure 1C). Similarly, the percentage of live cells for pioglitazone were found to be 94.19 ± 5.58% and 78.98 ± 3.00% in pretreated and post-treated cells, respectively. For further analysis, drug treatment prior to MPP^+^ exposure was chosen due to their similar response on cell viability when compared to post-treatment.

### 2.2. ROS Production and MMP Were Altered by Tideglusib or Pioglitazone Treatment in MPP^+^-Treated Cells

**Figure 2:**
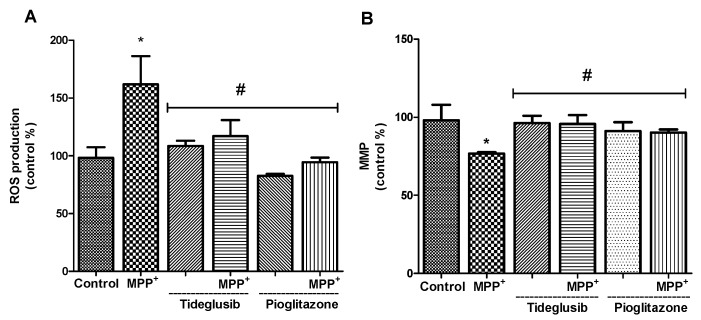
Effects of tideglusib or pioglitazone treatments in the presence or absence of MPP^+^ on reactive oxygen species (ROS) production and mitochondrial membrane potential (MMP) in SH-SY5Y cells. Cells were pretreated with tideglusib (2.5 µM) or pioglitazone (5 µM) for 1 h and exposed to MPP^+^ (2 mM) for 24 h. (**A**) Intracellular ROS accumulation was assayed using DCF-DA fluorescent dye. (**B**) MMP was measured using MitoTracker Red CMXRos staining. * *p* ˂ 0.001 vs. untreated cells, ^#^ *p* ˂ 0.05 vs. MPP^+^-treated cells.

Mitochondrial membrane potential and intracellular ROS were measured in order to demonstrate the link between MPP^+^ and oxidative stress and to assess the effects of tideglusib and pioglitazone on free radical production and mitochondrial dysfunction. As expected, MPP^+^ treatment significantly increased free radical production and decreased MMP when compared to untreated cells (*p* < 0.001) (Figure 2). Tideglusib and pioglitazone treatments reversed the effect of MPP^+^ on free radical production. Among these two drugs, pioglitazone was found to be more effective in reducing ROS production (*p* < 0.05). Regarding MMP, tideglusib pretreatment reversed the decrease in MMP from 76.73 ± 0.83% to 95.64 ± 5.73%, *p* ˂ 0.05 (Figure 2B). Similarly, pioglitazone increased the membrane potential to 90.23 ± 1.97% against MPP^+^.

### 2.3. Total GSH Levels and GST Enzyme Activity Were Regulated by Tideglusib in MPP^+^-Treated Cells

**Figure 3:**
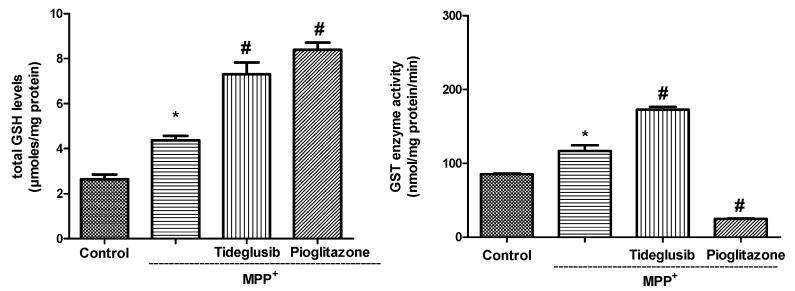
The changes in total glutathione (GSH) levels and glutathione-S-transferase (GST) enzyme activity in the presence or absence of tideglusib or pioglitazone in MPP^+^-treated cells. Cells were pretreated with tideglusib (2.5 µM) or pioglitazone (5 µM) for 1 h and exposed to MPP^+^ (2 mM) for 24 h. * *p* ˂ 0.05 vs. untreated cells, ^#^ *p* ˂ 0.05 vs. MPP^+^-treated cells.

To verify the effect of drugs on glutathione (GSH) levels, as an indicator of endogenous antioxidant system, and GST in MPP^+^-treated cells, we measured the changes in response to drug exposures (Figure 3). In tideglusib or pioglitazone-treated cells, the changes in total GSH levels showed similar patterns. Namely, pretreatment with drugs significantly increased total GSH levels when compared to untreated or MPP^+^-treated cells (*p* ˂ 0.05). GST enzyme activities were significantly increased in MPP^+^- and tideglusib-treated cells whereas pioglitazone pretreatment was found to decrease enzyme activity.

### 2.4. GSK-3β and PPARγ Levels Were Regulated by Tideglusib in MPP^+^-Treated Cells

**Figure 4:**
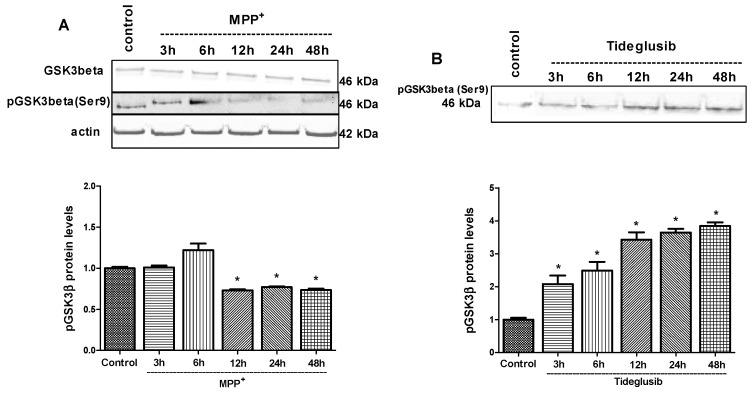
The changes in total GSK-3β and pGSK-3β (Ser9) following (**A**) MPP^+^ or (**B**) tideglusib treatments at indicated time points. Cells were treated with tideglusib (2.5 µM) or MPP^+^ (2 mM) for 3, 6, 12, 24, and 48 h. Protein expressions were measured by Western Blot analyses. Representative Western blots and graphs indicate the relative densitometric values of total GSK-3β and pGSK-3β (Ser9). Quantification of protein product was performed by densitometric scanning. Data are normalized by using the β-actin signal and expressed as arbitrary densitometric units. * *p* ≤ 0.001 vs. untreated cells.

The effect of MPP^+^ and tideglusib on the phosphorylation of GSK-3β at Ser9 was measured at 3, 6, 12, 24, and 48 h (Figure 4). MPP^+^ caused a significant decrease in pGSK-3β (Ser9) protein levels, which is an inactive form of the enzyme, at 12, 24, and 48 h, suggesting that MPP^+^ significantly triggered enzyme activation (*p* ≤ 0.001). On the other hand, the fold changes in pGSK-3β (Ser9) levels were found to increase to 2.08, 2.49, 3.42, 3.64, and 3.84 at 3, 6, 12, 24, and 48 h, respectively, following tideglusib treatment, confirming its inhibitory role in GSK-3β activity (Figure 4B).

**Figure 5:**
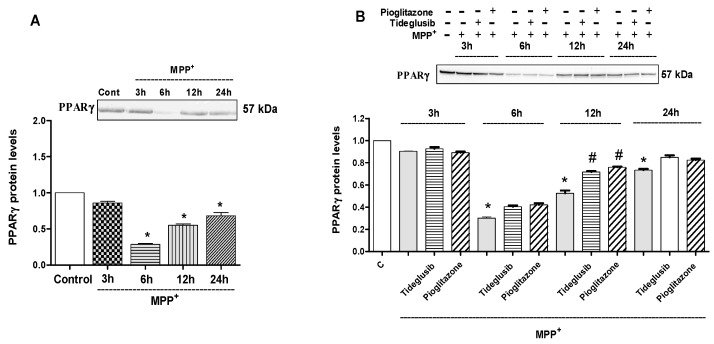
The changes in PPARγ protein levels following MPP^+^ and tideglusib or pioglitazone at different time points. (**A**) Cells were treated with MPP^+^ (2 mM) for 3, 6, 12, and 24 h. (**B**) Cells were pretreated with tideglusib (2.5 µM) or pioglitazone (5 µM) for 1 h and exposed to MPP^+^ for 3, 6, 12, and 24 h. The expressions of protein were measured by Western Blot analyses. Representative Western blots and graphs indicated the relative densitometric values of PPARγ. Quantification of protein product was performed by densitometric scanning. Data were normalized by using Lamin A signal and expressed as arbitrary densitometric units. * *p* ≤ 0.001 vs. untreated cells, ^#^ *p* ≤ 0.05 vs. MPP^+^-treated cells.

The effects of MPP^+^, tideglusib and pioglitazone on PPARγ protein levels were examined after 3, 6, 12, and 24 h of exposure (Figure 5). A 0.14 (*p* ˃ 0.05), 0.71, 0.45, and 0.32-fold decrease in expression of PPARγ were found at 3, 6, 12, and 24 h, respectively, in MPP^+^-treated cells (*p* ≤ 0.001). However, significant increases in PPARγ levels were observed at 12 h following tideglusib or pioglitazone treatments against MPP^+^ when compared to MPP^+^-treated cells (*p* ≤ 0.05).

### 2.5. Nrf2 and Nrf2-Related Proteins Were Regulated at Transcriptional and Translational Levels by Tideglusib

**Figure 6:**
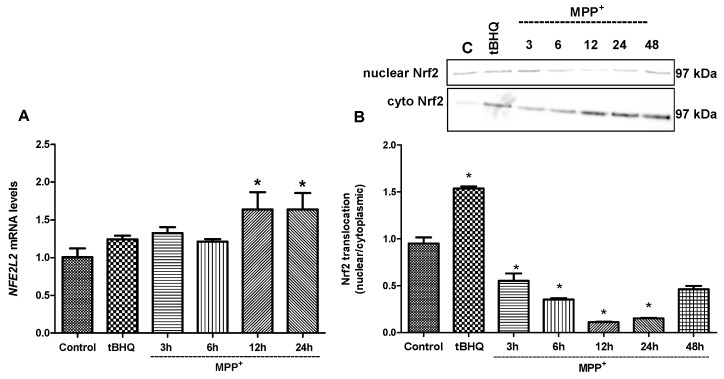
Time course experiments to monitor *NFE2L2* mRNA and Nrf2 nuclear/cytoplasmic protein levels after MPP^+^ exposure at indicated time points. Cells were treated with MPP^+^ (2 mM) for 3, 6, 12, 24, and 48 h or with tBHQ (100 µM) for 3 h. (**A**) Bar graph indicates the relative expression levels of Nrf2 mRNA. (**B**) Both cytosolic and nuclear protein extracts were prepared for Western blot analysis. Quantification of protein product was performed by densitometric scanning. Data are normalized by using the β-actin signal for cytosolic Nrf2 and Lamin A for nuclear Nrf2 and expressed as arbitrary densitometric units. * *p* ≤ 0.01 vs. untreated cells.

The effect of MPP^+^ on *NFE2L2* gene expression was determined using Real-time PCR at 3, 6, 12, and 24 h (Figure 6A). *NFE2L2* mRNA levels were increased 1.32-, 1.21-, 1.64, and 1.63-fold at 3, 6, 12, and 24 h, respectively, following MPP^+^ treatment when compared to untreated cells. Following MPP^+^ exposure, a significant increase in *NFE2L2* gene expression was observed by 12 h (*p* < 0.01). On the other hand, 100 µM tert-butylhydroquinone (tBHQ) a widely used Nrf2 activator, increased *NFE2L2* mRNA levels by 1.24-fold at 3 h. The concentration and time chosen for tBHQ in the present study was based on the efficacy of tBHQ as established in cell culture systems by other investigators [21,22].

In this study, the determination of Nrf2 translocation from the cytosol to the nucleus was used as a functional correlate for Nrf2 induction. As shown in Figure 6B, nuclear Nrf2 levels were decreased with MPP^+^ exposure in a time-dependent manner, whereas cytoplasmic Nrf2 levels were increased (*p* ≤ 0.01). A dramatic decrease in the amount of Nrf2 translocated to the nucleus at 12 h (0.11-fold, *p* ≤ 0.01) was observed when compared to untreated and MPP^+^-treated cells. Thus, the time required for the inhibition of nuclear translocation of Nrf2 was determined as 12 h for MPP^+^ treatment. SH-SY5Y cells treated with 100 μM tBHQ alone showed a 1.55-fold increase in Nrf2 protein translocation when compared to untreated cells.

**Figure 7:**
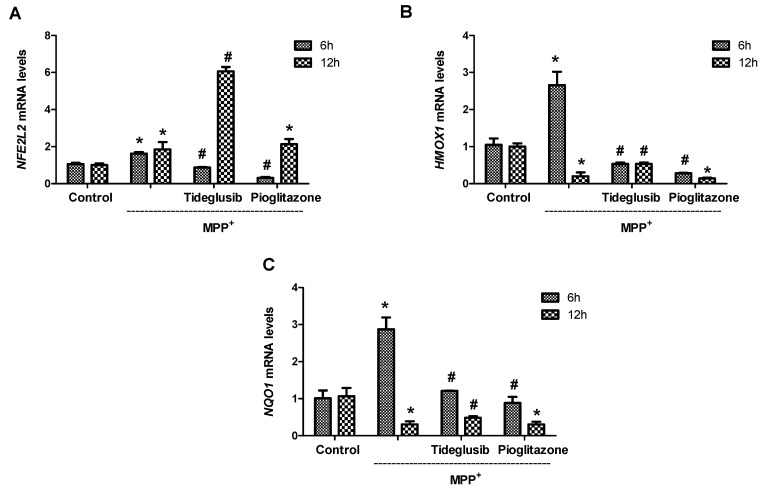
Time course experiments to monitor (**A**) *NFE2L2*, (**B**) *HMOX1*, and (**C**) *NQO1* mRNA levels in the presence or absence of tideglusib or pioglitazone following MPP^+^ treatment at 6 and 12 h. Cells were pretreated with tideglusib (2.5 µM) or pioglitazone (5 µM) for 1 h and exposed to MPP^+^ for 6 and 12 h. Bar graph indicated the relative mRNA expression levels of *NFE2L2*, *HMOX1*, and *NQO1*. * *p* ≤ 0.01 vs. untreated cell, ^#^ *p* ≤ 0.05 vs. MPP^+^-treated cells.

Following the determination of time point (12 h) when MPP^+^ caused a significant change in *NFE2L2* gene expression and Nrf2 nuclear translocation, drug-treated groups were established. In these groups, analyses of *NFE2L2*, *HMOX1*, and *NQO1* mRNA levels were performed at two different time points, including 6 and 12 h (Figure 7). It was found that MPP^+^ alone increased the expression of all three genes at 6 h (*NFE2L2* 1.62-fold, *HMOX1* 2.66-fold, *NQO1* 2.87-fold). On the other hand, tideglusib and pioglitazone treatments were found to reduce gene expressions at 6 h when compared to MPP^+^-treated cells (*p* ≤ 0.05). MPP^+^ decreased *HMOX1* and *NQO1* mRNA levels (0.19-fold for *HMOX1* and 0.30-fold for *NQO1*, respectively) (*p* ≤ 0.01), while increasing the *NFE2L2* mRNA levels (1.85-fold) at 12 h. In addition, the fold change in *NFE2L2* mRNA levels following tideglusib treatment was calculated as 3.29 (*p* ≤ 0.05) at 12 h when compared to MPP^+^-treated cells. Similarly, significant increases were found in *HMOX1* mRNA levels (0.53 ± 0.04) and *NQO1* mRNA levels (0.48 ± 0.04) after MPP^+^ treatment in the presence of tideglusib when compared to MPP^+^-treated cells at 12 h (*p* ≤ 0.05). However, no significant difference was found for all three genes in pioglitazone-treated cells at 12 h in comparison to MPP^+^ (*p* ˃ 0.05).

**Figure 8:**
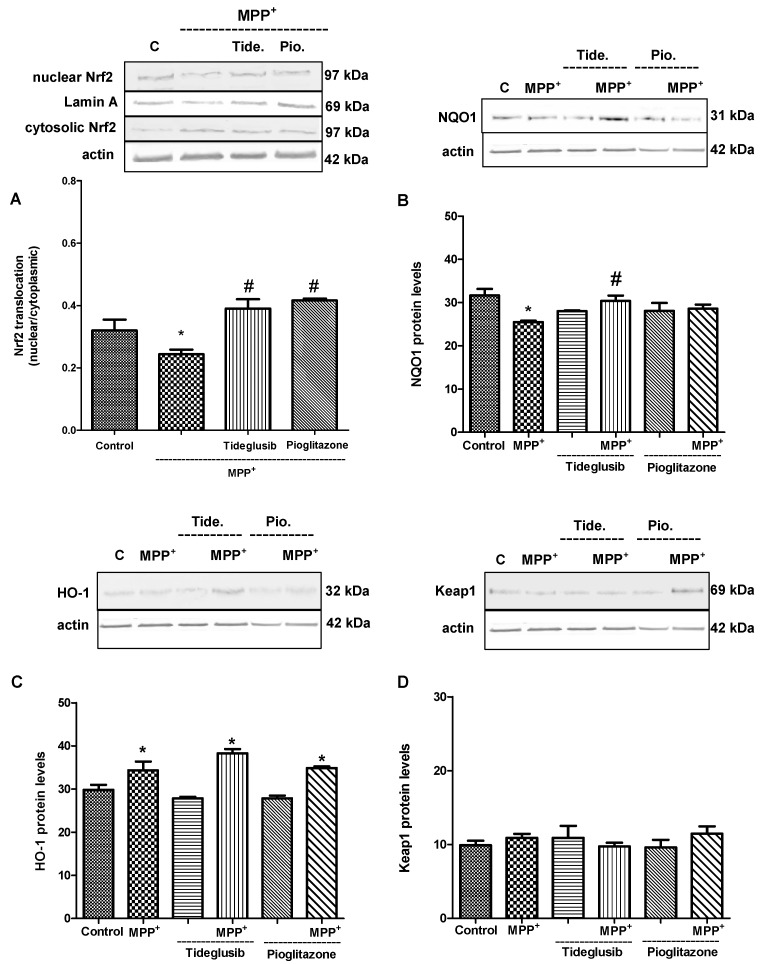
The changes in Nrf2 and Nrf2-related protein levels following MPP^+^ and tideglusib or pioglitazone. Cells were pretreated with tideglusib (2.5 µM) or pioglitazone (5 µM) for 1 h and exposed to MPP^+^ (2 mM) for 12 h. (**A**) Both cytosolic and nuclear protein extracts were prepared for Western blot analysis of Nrf2. (**B**) NQO1, (**C**) HO-1, and (**D**) Keap1 protein levels were determined in cytosolic fraction. Quantification of protein product was performed by densitometric scanning. Data are normalized by using the β-actin signal for cytosolic Nrf2, NQO1, HO-1, Keap1, and Lamin A for nuclear Nrf2 and expressed as arbitrary densitometric units. * *p* ≤ 0.01 vs. untreated cells, ^#^ *p* ≤ 0.05 vs. MPP^+^-treated cells.

The evaluation of the changes in Nrf2-related proteins such as HO-1, NQO1 can provide information about the antioxidant status of cell as well as Nrf2. Therefore, tideglusib or pioglitazone and MPP^+^ cotreated groups were designed after a period of time (12 h) when MPP^+^ caused significant decrease in Nrf2 nuclear translocation (Figure 8). We observed that both nuclear Nrf2 protein levels and nuclear translocation of Nrf2 were significantly promoted by drug administrations when compared to MPP^+^-treated cells (*p* ≤ 0.05). MPP^+^ exposure was found to reduce NQO1 protein levels (*p* ≤ 0.01). On the other hand, a significant increase in HO-1 levels (p ≤ 0.01) were observed with MPP^+^. NQO1 levels were increased after treatment with tideglusib when compared to MPP^+^ (*p* ≤ 0.05).

### 2.6. Nrf2 Silencing Reversed Tideglusib-Mediated Neuroprotection

**Figure 9:**
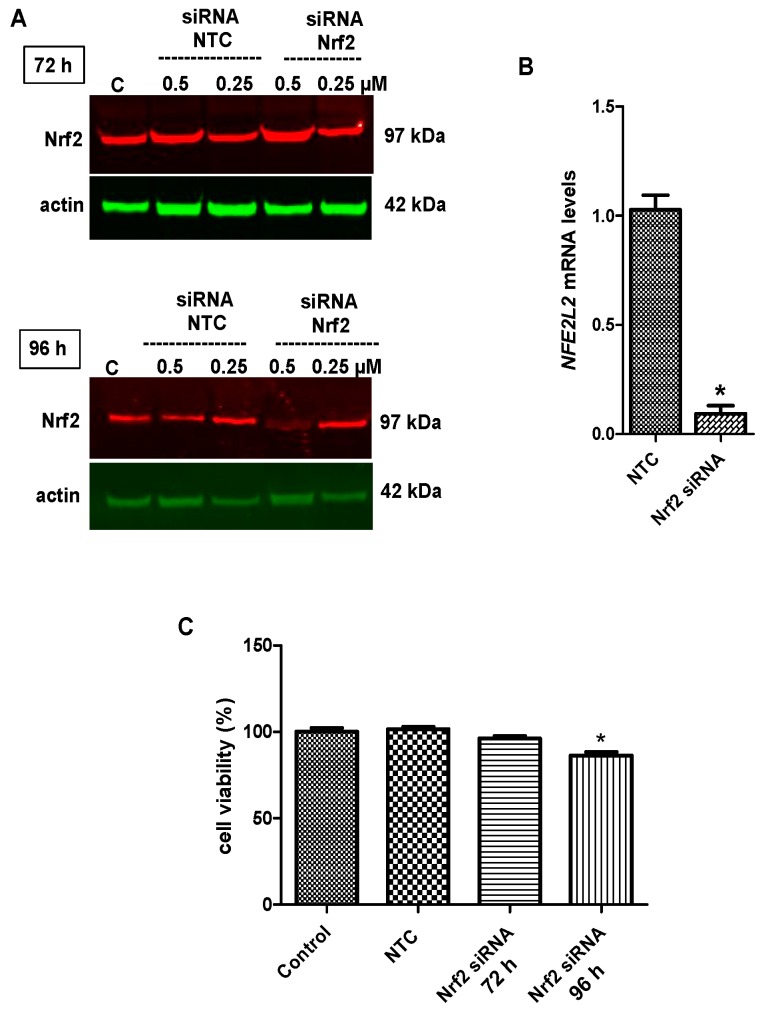
The confirmation of the knockdown efficiency of Nrf2 by Western Blotting, Real-time PCR and the effect of Nrf2 silencing on cell viability. (**A**) Cells were transfected with 0.25 or 0.5 µM siRNA targeting Nrf2 or negative control siRNA (NTC) for 72 or 96 h for fluorescent immunoblotting analysis. The expressions of protein were measured by Fluorescent Western Blot analyses in cytosolic fraction. (**B**) Cells were transfected with 0.5 µM siRNA targeting Nrf2 or NTC for 96 h for gene expression analysis. Bar graph indicated the relative mRNA expression levels of *NFE2L2*. (**C**) Cells were transfected with 0.5 µM siRNA targeting Nrf2 or NTC for 72 or 96 h for cell viability analysis. Cell viability was measured by MTT assay. * *p* < 0.05 vs. untreated cells.

To substantiate the role of Nrf2 in tideglusib-mediated neuroprotection, the effects of drugs on cell survival and mRNA/protein expressions of HO-1 and NQO1 were determined following introduction of Nrf2 siRNA into cells. At first, optimization studies were carried out to determine Nrf2 siRNA concentration and exposure time for the confirmation of siRNA assay. The knockdown efficiency of Nrf2 was determined by Real-time PCR and immunoblotting analyses and nearly 70% inhibition of Nrf2 levels were detected at 96 h (Figure 9A,B). Cell viability analysis was found similar to control group in Nrf2 siRNA group at 72 h (*p* ˃ 0.05). As expected, cell viability decreased by 13.68% (*p* < 0.01) at 96 h post-transfection (Figure 9C). Transfection with 0.5 µM concentration of Nrf2 siRNA for 96 h exposure were found to be sufficient for gene silencing in our experimental model.

**Figure 10:**
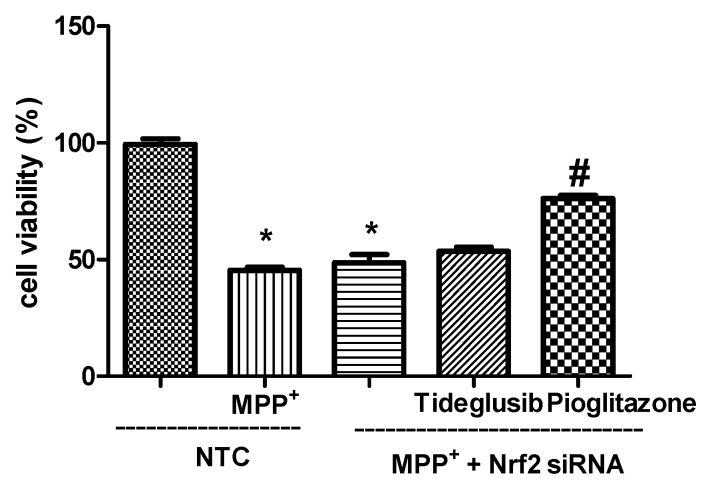
The changes in cell viability in Nrf2-siRNA transfected cells. Cells were transfected with 0.5 µM siRNA targeting Nrf2 or NTC for 96 h. The transfected cells were pretreated with tideglusib or pioglitazone for 1 h and exposed to MPP^+^ for 24 h. Cell viability was measured by MTT assay. * *p* < 0.05 vs. NTC only, ^#^ *p* < 0.05 vs. MPP^+^-treated cells.

To verify the requirement of Nrf2 for tideglusib-induced neuroprotection, cells were transfected with siRNA for 96 h before drug treatments. As shown in Figure 10, cell viability in Nrf2-siRNA transfected cells was found to be 42.37%, 53.56%, and 76.18% in MPP^+^-treated, MPP^+^ + tideglusib-treated and MPP^+^ + pioglitazone-treated cells, respectively (*p* < 0.05). Pioglitazone significantly increased cell viability in Nrf2-siRNA transfected cells. However, tideglusib failed to reverse the toxic effects of MPP^+^ when Nrf2 gene was silenced.

**Figure 11:**
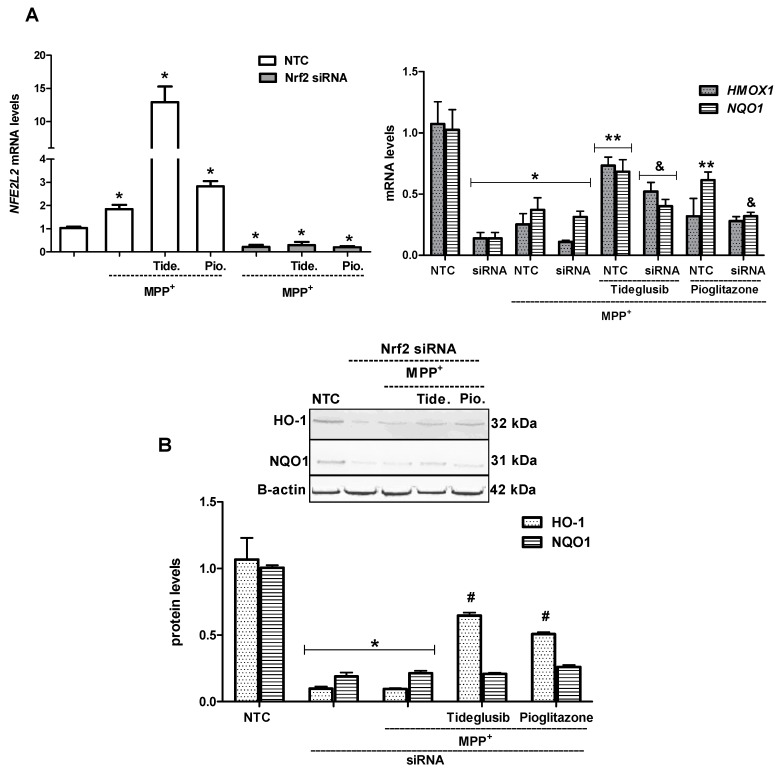
The changes in *NFE2L2* mRNA, *HMOX1* and *NQO1* mRNA/protein levels in Nrf2-siRNA transfected cells. Cells were transfected with 0.5 µM siRNA targeting Nrf2 or negative control siRNA (NTC) for 96 h. Both Nrf2-siRNA transfected and NTC cells were pretreated with tideglusib or pioglitazone for 1 h and exposed to MPP^+^ for 12 h. (**A**) Bar graph indicates the relative expression levels of *NFE2L2*, *HMOX1* and *NQO1* mRNA. (**B**) Protein expressions were measured by Western Blot analyses. Representative Western blots and graphs indicated the relative densitometric values of HO-1 and NQO1. Quantification of protein product was performed by densitometric scanning. Data are normalized by using the β-actin signal and expressed as arbitrary densitometric units. * *p* < 0.05 vs. NTC only, ** *p* < 0.05 vs. NTC+MPP^+^-treated cells, ^#^ *p* < 0.05 vs. MPP^+^+Nrf2 siRNA transfected cells, ^&^ *p* < 0.05 vs. drug-treated NTC cells.

Moreover, cells were transiently transfected with Nrf2-siRNA to evaluate the effects of drugs on downstream proteins in the absence of Nrf. mRNA levels of *HMOX1* and *NQO1* significantly decreased in Nrf2-siRNA and MPP^+^+Nrf2-siRNA treated cells when compared to NTC cells (*p* < 0.05). As expected, upregulation of *NFE2L2* or *HMOX1* and *NQO1* mRNA expressions by drug treatments against MPP^+^ was significantly abolished by siRNA-mediated knockdown of Nrf2 (Figure 11A). Similarly, when compared with the data obtained in the absence of Nrf2 siRNA, knockdown of Nrf2 significantly reversed increased protein levels of downstream proteins, particularly NQO1, in tideglusib or pioglitazone-treated cells against MPP^+^ (Figure 11B), suggesting that the protective effects of drugs are mediated through Nrf2 activation.

## 3. Discussion

The induction of endogenous antioxidant and protective mechanisms, including the Nrf2/ARE pathway, can be considered as a beneficial strategy for inhibiting oxidative stress. The available evidence reports that GSK-3β has a critical role in oxidative stress-mediated neuronal death. Therefore, this enzyme is considered as an important therapeutic target for the development of promising selective inhibitors in diseases [12,15]. Clinical efficacy and tolerability of tideglusib, an irreversible GSK-3β inhibitor, have been tested in patients with progressive supranuclear palsy [23]. Phase II studies are still under investigation for autism spectrum disorder and congenital and juvenile myotonic dystrophy. We aimed to evaluate the potential therapeutic role of tideglusib and to exert possible mechanisms involved in tideglusib-mediated neuroprotection in MPP^+^-induced neuronal damage since Parkinsonian agents such as MPP^+^, 6-hydroxydopamine (6-OHDA), and rotenone have been reported to induce neuronal damage via GSK-3β activation [24,25,26]. In our study, MPP^+^ treatment was shown to decrease phosphorylated GSK-3β (Ser9) levels as an indicator of enzyme activation (Figure 4A). In accordance with our results, Zhang et al. [27] reported that total GSK-3β mRNA and protein levels were increased by MPP^+^-mediated toxicity in SH-SY5Y cells.

In the current study, prophylactic (pretreatment) or therapeutic (post-treatment) approaches were aimed to determine whether there is any superiority. Pretreatment was found to be similar to post-treatment in terms of cellular protection (Figure 1). GSK-3β is overactivated in pathological conditions [9,10,11,12,13]. However, in physiological conditions, this enzyme regulates many and diverse cellular functions. It can be suggested that drugs used in this study inhibit enzyme at the moment the stimulation of enzyme begins by MPP^+^. Thus, the protection by GSK3β inhibitors may be observed in the presence of toxins and in situations when enzyme overactivated. As shown in Figure 1C, both pre- and posttreatment of tideglusib at 2.5 µM significantly reduced MPP^+^-mediated dopaminergic damage. Similar to our findings, in a study by Luna-Medina et al. [28] investigating the neuroprotective effects of tideglusib in excitotoxic conditions, 2.5 μM tideglusib against glutamate-mediated neurotoxicity has been shown to exert neuroprotection. Few GSK-3β inhibitor families, including the TDZD family, were reported to exert their action with non-ATP competitive mechanism. Thiadiazolidines are small molecules with favourable ADME-Tox drugable properties, such as oral bioavailability and blood–brain barrier penetration [16]. In addition, these molecules were shown to exhibit PPAR-gamma activating properties. Thiazolidinediones (TZDs) including pioglitazone have similar structure with TDZDs. Thus, inhibitory effects on GSK-3β are expected for TZDs similar to TDZDs. Pioglitazone was previously shown to regulate GSK-3β levels against cardiac hypertrophy in vivo [29]. For this reason, pioglitazone is expected to inhibit GSK-3β in our experimental model as well. On the other hand, in previous studies, treatment with a specific TDZD compound (i.e., tideglusib) was shown to decrease amyloid deposition, tau phosphorylation, and astrocytic proliferation and prevent memory deficits in transgenic mouse model [19]. These results support the effective role of tideglusib on neuronal survival when compared to TZDs.

Due to the structural similarity to TZDs, the modulatory role of TDZDs on PPARγ was previously investigated [13,17]. PPARγ agonists are one of the most promising classes of drugs that induce neuroprotection against inflammation, oxidative stress, and apoptosis [30,31,32]. In the light of these findings, exploring the mechanisms of action of tideglusib (including GSK-3β inhibition and PPARγ activation) are important in terms of neurodegenerative diseases. For this reason, while evaluating its possible dual role, tideglusib was compared with pioglitazone, a well-known PPARγ agonist, which was previously shown to be effective in Parkinson’s disease models. The effect of MPP^+^ on PPARγ protein levels was examined at different time points (3, 6, 12, and 24 h) and time-dependent decreases in receptor levels suggest that these receptors are affected by MPP^+^-mediated toxicity (Figure 5A). Significant increases in PPARγ following tideglusib or pioglitazone treatments against MPP^+^ confirmed their effect on PPARγ in our experimental model (Figure 5B).

Remarkable interest has risen in the idea that antioxidant mechanisms can slow the progressive loss of dopaminergic neurons. In the current study, tideglusib and pioglitazone were shown to reverse increased MPP^+^-induced ROS production, decreased antioxidant enzyme activation, and altered mitochondrial membrane potential (Figure 2 and Figure 3). Similar to our findings, Petit-Paitel et al. [26] reported that GSK-3β inhibition abolished MPP^+^-mediated mitochondrial membrane potential alteration. The first step of GSH biosynthesis is rate limiting and catalyzed by glutamate-cysteine ligase (GCL) which is composed of a catalytic (GCLC) and a modifier (GCLM) subunits. Nrf2 is known to bind and trans activate the ARE present in the human GCLC and GCLM promoters in response to its activation [33]. Although the levels of these subunits were not evaluated in our experimental model, they are likely upregulated by chosen drugs. As shown in Figure 3, treatment with tideglusib or pioglitazone resulted in modest elevation in GSH levels against MPP^+^, indicating that the regulatory role of these drugs on endogenous antioxidant systems may be a result of Nrf2 activation. Moreover, our unpublished data showing significant decrease in Bax/Bcl-2 protein ratio by tideglusib suggesting that tideglusib may also have antiapoptotic effects in MPP^+^-induced cellular damage.

Under oxidative stress conditions, such as H_2_O_2_, glutamate, dopamine, 6-OHDA, MPTP, tetrahydrobiopterin, and 3-morpholinosindnonimine-induced oxidative stress, activation of the Nrf2/ARE pathway by tBHQ or sulforaphane was shown to protect neuronal cell lines and primary cortical cultures [34,35,36,37,38]. Increased NQO1 and HO-1 levels shown in postmortem brain tissues of Parkinson’s disease patients as well as increased nuclear levels of Nrf2 suggest that induction of expression of Nrf2-regulated proteins could be an important survival mechanism [39,40]. In our study model, both Nrf2 and Nrf2-related gene expression levels were determined following treatments. As shown in Figure 6, MPP^+^ increased *NFE2L2* mRNA levels in a time-dependent manner. Our data suggest that *NFE2L2* expression is not only regulated at the transcriptional level, but also at a level of protein synthesis by MPP^+^. HO-1 synthesis has been reported to increase in brain cells under stress conditions, indicating that HO-1 plays an important role in response to stress [41]. In the present study, obtained data at earlier time point (6 h) demonstrating increased expressions of downstream genes following MPP^+^ indicate that key compensatory protective mechanisms during the early stages of oxidative insult are activated (Figure 7). It is noteworthy that while MPP^+^ increased *NFE2L2* mRNA levels, it decreased *HMOX1* and *NQO1* mRNA levels in time. This leads to the idea that by the time of cellular damage, nuclear import of Nrf2 might be impaired by MPP^+^-triggered intracellular mechanisms or Nrf2 might be degraded in the nucleus before binding to the DNA binding domain before triggering protective gene expression. We confirmed this idea with the data showing a decrease in nuclear translocation of Nrf2 following MPP^+^ at 12 h (Figure 8A).

The protein analyses of the mentioned genes were performed simultaneously to support the obtained data in gene expression analysis. In accordance with previous findings [42], we showed that MPP^+^ significantly reduced nuclear Nrf2 and NQO1 protein levels (Figure 8). Tideglusib and pioglitazone treatments achieved to increase Nrf2 translocation against MPP^+^ (Figure 8). These observations suggest that both agents may play active roles in controlling the translocation of Nrf2 from cytoplasm to nucleus and/or the duration of Nrf2 in nucleus. Overexpression of HO-1 was shown to protect dopaminergic neurons against MPP^+^-induced neurotoxicity [43]. Similarly, it has been reported induction of NQO1 leads to protection of dopaminergic cells in vitro [44,45]. Thus, the achievement of studied drugs in increasing HO-1 and NQO1 protein levels similar to untreated group is another indication that these agents have regulatory role in Nrf2 pathway while protecting cells. In addition, as shown in Figure 7, increases in *HMOX1*, *NQO1*, and *NFE2L2* gene expressions in a period of time (at 12 h) may be a result of triggering antioxidant systems through GSK-3β inhibition. As mentioned before, the activation of GSK-3β following MPP^+^ was not observed before 12 h exposure. Probably for this reason, we could not observe positively regulatory role of tideglusib on Nrf2-related gene expressions at earlier time point.

GSK-3β exerts a negative form of regulation on Nrf2 by controlling it is subcellular distribution [6,46]. Although our findings regarding to triggering Nrf2 nuclear translocation and consequently induction of Nrf2-related gene/protein expressions (HO-1, NQO1) by GSK-3β inhibition in MPP^+^-induced cellular death supported our hypothesis, cells were transiently transfected with Nrf2-siRNA to clarify whether tideglusib-induced expression of HO-1 and NQO1 proteins were mediated by Nrf2 activation. In particular, decreased efficiency of tideglusib in presence of Nrf2 silencing on cell viability in MPP^+^-induced cellular damage is evidence that Nrf2 signaling is critical for tideglusib-mediated neuroprotection (Figure 10). Conversely, cellular protection by pioglitazone was not affected under the same condition indicating that pioglitazone may trigger other protective mechanisms in addition Nrf2. Although tideglusib showed a tendency to upregulate *HMOX1* and *NQO1* gene expressions in presence of Nrf2 in MPP^+^-induced toxicity, Nrf2 silencing significantly influenced its regulatory role on gene expressions (Figure 11A). Similar observations obtained for *NQO1* mRNA levels following pioglitazone treatment indicate the possible role of Nrf2 in drugs-induced downstream gene expression. As shown in Figure 11B, Western blot results showed that following Nrf2 gene silencing, both tideglusib and pioglitazone against MPP^+^ increased HO-1 levels but did not cause significant changes in NQO1 levels. This suggests that tideglusib exerts its regulatory role particularly on NQO1 protein synthesis while regulating Nrf2. On the other hand, the studied drugs may utilize different mechanisms independent of Nrf2 in the regulation of HO-1 gene expression. HO-1 mRNA synthesis is known to be induced by PPARγ receptor activation in human vascular endothelial and smooth muscle cells [47]. Thus, the major reason for elevated HO-1 levels following drug treatments in Nrf2 silenced cells may be due to their effects on PPARγ rather than GSK3β. This should be elucidated in further studies. Clearly, activation of detoxifying enzymes and protective proteins through the Nrf2–Keap1 system could counteract MPP^+^-mediated toxicity. The effects of tideglusib against MPP^+^-induced cell death and cytotoxicity were abrogated by transfection of cells with Nrf2-siRNA, suggesting that tideglusib-induced protection is mediated through Nrf2 activation.

## 4. Materials and Methods

### 4.1. Cell Culture and Treatments

The human neuroblastoma cell line (SH-SY5Y) used in this study was purchased from the American Type Culture Collection (ATCC, Catalog #CRL-2266). Cells were suspended in complete Dulbecco’s modified Eagle Medium (DMEM) (Life Technologies, Gibco BRL, Grand Island, NY) supplemented with 10% fetal bovine serum (FBS, Hyclone), 1% penicillin and streptomycin (100 U/mL, Invitrogen) and plated in cell culture dishes. The cultures were maintained at 37 °C in 5% CO_2_ 95% humidified atmosphere. After reaching 85% confluence, cells were transferred to 96-well plate or culture dish and allowed to adhere for 24 h. The culture medium was replaced with fresh medium containing compounds (tideglusib or pioglitazone) and/or 2 mM MPP^+^ and incubated in 5% CO_2_ incubator at different time points. Tideglusib (0.1–50 µM) and pioglitazone (0.1–15 µM) in various concentrations dissolved in dimethyl sulfoxide (DMSO) were added and incubated at 37 °C in 5% CO_2_ for 3, 6, and 24 h for cell proliferation assay. The concentration used for MPP^+^ was determined in our previous study and adopted to present study [20]. The final concentration of DMSO in cell culture was less than 0.2%. The concentrations of tideglusib used in the present study were based on the efficacy of tideglusib as established in cell culture systems by our laboratory and other investigators [13,28].

For 3-(4,5-dimethylthiazol-2-yl)-2,5-diphenyl tetrazolium bromide (MTT), ROS, and MMP experiments, cells were seeded into 96-well cell culture plates at a density of 2 × 10^3^ cells per well. For enzyme activities and protein analysis, cells were seeded into 6-well cell culture plates at a density of 5 × 10^5^ cells/well. When used, all compounds were added 1 h prior to MPP^+^ unless otherwise noted.

### 4.2. Cell Viability Assessment

Percent cell viability was assessed using the MTT assay. Briefly, following exposures, MTT (in final concentration 0.5 mg/mL) was added to each well, and plates were incubated for 3 h at 37 °C in 5% CO_2_ humidified incubator. The reaction mixture was carefully removed and 200 µL DMSO was added to each well. The plate was shaken at room temperature for 1 h and then the absorbance was measured at 570 nm and 630 nm using a microplate reader (VersaMax, Molecular Devices, USA). Percent survival was plotted relative to vehicle control cells, which were normalized to 100% survival.

### 4.3. Measurement of ROS Production

Intracellular ROS generation was measured using ROS sensitive cell permeable fluorescent dye 2′,7′-dichlorodihydrofluorescein diacetate (H_2_DCF-DA) as previously described [13]. The changes in ROS production in response to different treatments were estimated by fluorescence intensity, with excitation wavelenght at 485 nm and emission wavelength at 535 nm using Varioskan Flash Multimode Reader (Thermo Scientific, Vantaa, Finland). ROS production was expressed as a percentage of control cells.

### 4.4. Analysis of MMP

Mitochondrial function was assessed by using MitoTracker Red CMXRos (Molecular Probes, Eugene, OR) staining, an indicator of MMP. Briefly, following exposures, cells were loaded with 50 nM MitoTracker Red CMXRos during the last 30 min of treatment at 37 °C before fixing (4% para-formaldehyde) for microscopy. Slides were analyzed by fluorescence microscopy. Images were captured at 40 magnification using an Olympus BX3 (Tokyo, Japan) fluorescence microscope. Corrected total cell fluorescence was calculated as follows: CTCF = Integrated Density–(Area of selected cell 9 Mean fluorescence of background readings) using Image J software (National Institutes of Health, Bethesda, MD, USA). This analysis was performed in at least 60 cells per measurement.

### 4.5. Total Glutathione Levels

The levels of total glutathione were measured using colorimetric GSH assay kit (Bioassay). This kit utilizes an optimized enzymatic GR recycling method for quantification of GSH. Briefly, GSH is oxidized by 5,5′-dithiobis-(2-nitrobenzoic acid) (DTNB) resulting in the formation of GSSG and 5-thio-2-nitrobenzoic acid (TNB). GSSG is then reduced to GSH by glutathione reductase (GR) using reducing equivalent provided by NADPH. The rate of TNB formation is proportional to the sum of GSH and GSSG present in the sample and is determined by measuring the formation of TNB at 412 nm. Each sample was assessed in duplicates, and the levels of total GSH were expressed as µmoles/mg protein.

### 4.6. GST Enzyme Activity

Enzyme activity was determined using GST assay kit (Bioassay). This kit measured total GST activity (cytosolic and microsomal) using 1-chloro-2,4-dinitrobenzene (CDNB) as the substrate. Briefly, the reaction was initiated by addition of 10 μL of 20 mM CDNB to the reaction mixture (200 μL) contained 20 μL of homogenate, 100 mM potassium phosphate (pH 6.5), 0.1% (*v*/*v*) Triton X-100, 5.0 mM reduced GSH at room temperature. The increase in absorbance at 340 nm was recorded at 60 s intervals for 7 min. The rate of increase in the absorbance is directly proportional to the GST activity in the sample. Each assay was performed in duplicate, and enzyme units were recorded as nmol/min/mg protein.

### 4.7. Total RNA Isolation, Reverse Transcription PCR, and Real-Time PCR

Total RNA was extracted using the MasterPure RNA Purification Kit (Epicentre Biotechnologies). The concentration of RNA in each sample was measured at 260 nm, and its integrity was assessed after electrophoresis (data not shown). Prior to the reverse transcription reaction, potentially contaminating residual genomic DNA was eliminated with DNAse I (MBI Fermentas). 1 µg/µL total RNA was used for first strand complementary DNA (cDNA) synthesis by MMuLV reverse transcriptase (MBI Fermentas). *NFE2L2 (Nrf2*, GenBank ID: NM_006164), *NQO1* (GenBank ID: NM_000903), *HMOX1 (HO-1*, GenBank ID: X06985) and *glyceraldehyde-3-phosphate dehydrogenase* (*GAPDH*) (GenBank ID: NM_002046) primers were newly designed using Primer3 software [48]. The forward and reverse primers were shown in Table 1. Conditions for PCRs were optimized in a gradient cycler with regard to primers and various annealing temperatures. Amplified cDNA samples were assessed by Real-time PCR (LightCycler^®^ 480 System, Roche). Both cDNA synthesis and PCR amplifications included negative control reactions, which were set up by excluding RNA and DNA templates, respectively. The amplification specificity of the PCR products of *NFE2L2*, *NQO1* and *HMOX1* were confirmed by the melting curve analysis (data not shown). *GAPDH* gene was used as an endogenous control for normalization. The relative expressions of target genes were quantified according to ABI Prism 7700 Sequence Detection System User Bulletin No. 2 (Applied Biosystems, Foster City, CA, USA) and Schmittengen and Livak [49]. The relative expression levels (fold changes) of mRNAs were calculated by 2^−ΔΔCT^ method. Real-time PCR products were normalized to its corresponding *GAPDH* mRNA.

### 4.8. Nrf2 siRNA Transfection

SH-SY5Y cells were transiently transfected with siRNA targeting to Nrf2 by Accell siRNA delivery media (Dharmacon) according to the manufacturer’s protocol. Briefly, 1.5 × 10^5^ cells were plated into a 12 well plate and incubated at 37 °C with 5% CO_2_ overnight. 0.25 or 0.5 µM final concentrations of Nrf2 siRNA (Smartpool, Dharmacon) in 1×siRNA buffer diluted from 5×siRNA buffer (Dharmacon) was added to the wells. The cells were incubated at 37 °C with 5% CO_2_ for 72 and 96 h. The knockdown efficiency of Nrf2 was determined at 0.5 µM concentration of Nrf2 siRNA at 96 h post-transfection with immunoblotting. Following the transfection, the cells were treated by 2 mM MPP^+^ for 12 h or 24 h with or without tideglusib or pioglitazone. Then, the cells were used for cell viability, gene and protein expression analyses.

### 4.9. Isolation of Cytoplasmic and Nuclear Fraction

The cytoplasmic and nuclear fractions were prepared using NE-PER Nuclear and Cytoplasmic Extraction Reagents Kit (Thermo Scientific, Waltham, MA), following the manufacturer’s instruction.

### 4.10. Western Blotting

Western blotting was performed by loading 30 μg protein on 8–12% (*w*/*v*) tris–glycine denaturing gels and separating proteins by electrophoresis, then transferring to PVDF membrane. After blocking, the membrane was incubated with primary antibodies; anti-Nrf2 rabbit monoclonal antibody (1:1000, Cell Signaling Technology), anti-HO-1 rabbit monoclonal antibody (1:1000, Cell Signaling Technology), anti-NQO1 rabbit monoclonal antibody (1:1000, Cell Signaling Technology), anti-pGSK-3β (Ser9) rabbit monoclonal antibody (1:1000, Cell Signaling Technology), anti-GSK-3β rabbit monoclonal antibody (1:1000, Cell Signaling Technology), anti-PPARγ rabbit monoclonal antibody (1:1000, Cell Signaling Technology), anti-Keap1 rabbit monoclonal antibody (1:1000, Cell Signaling Technology) at +4 °C over night. Following the incubation, the membrane was incubated with anti-β-actin mouse monoclonal antibody (1:1000, Cell Signaling Technology) or anti-lamin A mouse monoclonal antibody (1:1000, Cell Signaling Technology) at room temperature for 1 h. After washing, the membrane was incubated with peroxidase-conjugated secondary antibodies for 1 h to visualize labeled proteins by enhanced chemiluminescence.

To confirm efficient knockdown of Nrf2 expression by siRNA transfection, Nrf2 protein expression levels were also detected using LI-COR IRDye infrared dye secondary antibodies and visualized using an Odyssey infrared imager (LI-COR Biotechnology).

### 4.11. Statistical Analysis

All data were expressed as means ± standard deviation (SD) for six independent experiments for MTT/ROS/MMP analyses and three independent experiments for gene/protein expression analyses. Comparisons of means between groups were performed by one-way analysis of variance (ANOVA) followed by Tukey’s post hoc test. *p* < 0.05 was considered statistically significant.

## 5. Conclusions

A key implication of these results is the possibility that GSK-3β inhibitors, such as tideglusib, offer an alternative to targeting oxidative stress that offers advantages including absorption, distribution, metabolism, excretion, and toxicity properties such as oral bioavailability and blood–brain barrier penetration when compared to several antioxidant molecules. The activation of Nrf2 is considered to be one of the mechanisms that reduce the detrimental effects of oxidative stress. Moreover, cell protection mechanisms are thought to be more effective when Nrf2 activation is triggered. In particular, subcellular localization of Nrf2 and changes in the expression of antioxidant genes suggest that surviving cells try to benefit from the Nrf2-mediated protective response. Nuclear translocation of Nrf2 is very important in terms of efficacy. The subcellular localization of Nrf2 is regulated by Keap1 mediated ubiquitination and phosphorylation. However, drug studies investigating the importance of phosphorylation in the regulation of Nrf2 are limited. One of the primary goals of this study was to demonstrate the neuroprotective potential of a kinase inhibitor and the relevance of Nrf2 signaling for protection of tideglusib was shown in MPP^+^-induced cellular damage. From a clinical point of view, pretreatment is very difficult in practice. Besides, preservation of existing cells by Nrf2 activators after injury suggests that such agents are promising both in terms of pre- and postapplications. In further studies, pre- and postinjury applications in animal and/or clinical trials while evaluating the efficacy of drugs need to be elucidated.

## Figures and Tables

**Table 1:** Sequences of forward and reverse primers used for PCR analysis.

Gene	Forward Primer (5′→3′)	Reverse Primer (5′→3′)	Base Pair
NFE2L2 (NM_006164)	agc gac gga aag agt atg ag	tgg gca acc tgg gag tag	192 bp
NQO1 (NM_000903)	cag ctc acc gag agc cta gt	gag tga gcc agt acg atc agt g	122 bp
HMOX1 (X06985)	cca gcg ggc cag caa caa agt gc	aag cct tca gtg ccc acg gta agg	265 bp
GAPDH (NM_002046)	agc cac atc gct cag aca c	gcc caa tac gac caa atc c	65 bp
